# Microbial Biofilms: Human T-cell Leukemia Virus Type 1 First in Line for Viral Biofilm but Far Behind Bacterial Biofilms

**DOI:** 10.3389/fmicb.2020.02041

**Published:** 2020-09-15

**Authors:** Yousef Maali, Chloé Journo, Renaud Mahieux, Hélène Dutartre

**Affiliations:** CIRI - Centre International de Recherche en Infectiologie, Univ Lyon, Université Claude Bernard Lyon 1, Inserm, U1111, CNRS, UMR5308, ENS Lyon, Lyon, France

**Keywords:** human T-cell leukemia virus type 1, viral biofilm, extracellular matrix, cell-cell transmission, pathogenesis

## Abstract

Human T-cell leukemia virus type 1 (HTLV-1) is a retrovirus associated with adult T-cell leukemia (ATL) and HTLV-1-associated myelopathy/tropical spastic paraparesis (HAM/TSP). To date, it is the unique published example of a virus able to form a biofilm at the surface of infected cells. Deeply studied in bacteria, bacterial biofilms represent multicellular assemblies of bacteria in contact with a surface and shielded by the extracellular matrix (ECM). Microbial lifestyle in biofilms, either viral or bacterial, is opposed structurally and physiologically to an isolated lifestyle, in which viruses or bacteria freely float in their environment. HTLV-1 biofilm formation is believed to be promoted by viral proteins, mainly Tax, through remodeling of the ECM of the infected cells. HTLV-1 biofilm has been linked to cell-to-cell transmission of the virus. However, in comparison to bacterial biofilms, very little is known on kinetics of viral biofilm formation or dissemination, but also on its pathophysiological roles, such as escape from immune detection or therapeutic strategies, as well as promotion of leukemogenesis. The switch between production of cell-free isolated virions and cell-associated viral biofilm, although not fully apprehended yet, remains a key step to understand HTLV-1 infection and pathogenesis.

## Introduction

The extracellular matrix (ECM), defined as the three-dimensional network of high-molecular-weight extracellular molecules that surround the surface of the cells that produce them, is one of the few structures conserved during evolution and shared through the three biological domains of life: *Archaea*, Bacteria, and Eukarya (Stavolone and Lionetti, 2017). Although its composition varies according to the type of organism, i.e., prokaryote vs. eukaryote organisms, or unicellular vs. multicellular organisms, the ECM remains required for conserved functions, such as cell adhesion, intercellular communication, and differentiation (Ozbek et al., 2010; Limoli et al., 2015).

In bacteria, the ECM consists of three major polymer components: (i) polysaccharides, (ii) proteins, and (iii) extracellular DNA (eDNA). Bacterial ECM may serve several functions, such as protection of free-living bacteria (planktonic life), promotion of migration or genetic exchanges, and ion reservoirs. In addition, the ECM is an essential building material for biofilm formation (Dragoš and Kovács, 2017). Bacterial biofilms (sessile life) represent multicellular assemblies, in contact with a surface, in which bacteria stick to each other and are shielded by the ECM. They represent the most ubiquitous bacterial lifestyle in natural and artificial environments.

The eukaryotic ECM, whether in tissues or on circulating cells, is a network of extracellular macromolecules made up of core proteins (collagens, glycoproteins, and proteoglycans) and of ECM-associated components, including adhesion proteins and cellular receptors (Mouw et al., 2014). The ECM serves as a physical scaffold to the cell but it is also a dynamic structure interacting with other cells to regulate diverse cellular functions, including survival, growth, migration, and differentiation, as well as response to diseases (Bellincampi et al., 2014; Bonnans et al., 2014; Humphrey et al., 2014). Interestingly, the ECM can be strongly remodeled early upon viral infection. Viruses can use the ECM for attachment to target cells and subsequent interactions with cell-surface receptors allowing their entry. Besides its role to favor initial infection, modulation of the ECM from infected cells can impact viral disease manifestation directly or indirectly. For example, modification of the ECM by viruses may modulate inflammation. This is observed upon respiratory syncytial virus (RSV) infection of fibroblasts *via* hyaluronan-dependent mechanisms, which enhances mast cell binding as well as mast cell protease expression *via* direct interactions with the ECM (Reeves et al., 2020). It can also influence the process of carcinogenesis. For instance, Hepatitis B virus (HBV) encodes a viral onco-protein, transactivator protein X, involved in the ECM remodeling through the HIF-1α/LOX pathway, which is shown to promote hepatocellular carcinoma metastasis (Tse et al., 2018). After productive infection, modulation of the ECM can also enhance viral transmission through cell-to-cell contact. Indeed, infected cells can establish contacts between cells that are not normally in physical contact using for example, increased migration of infected cells toward non-infected cells. Alternatively, infected cells can exploit existing cell-cell interactions, such as immunological synapses, to increase adhesiveness and viral transfer through the immune synapse. For that purpose, some viruses such as human T-cell leukemia virus type 1 (HTLV-1) can upregulate the expression of endogenous cell adhesion molecules (CAM), such as Intercellular adhesion molecule-1 (ICAM-1), and of other components of the ECM (Nakachi et al., 2011; Gross and Thoma-Kress, 2016), while others can produce their own adhesion proteins. For example, the glycoprotein Env from Murine Leukemia Virus (MLV) can act as a viral adhesion molecule (VAM), mimicking the behavior of a CAM (Sherer et al., 2007; Mothes et al., 2010). Moreover, these virally induced areas of contact can be highly specialized and fully dedicated to viral transmission, as exemplified by the ability of herpes simplex virus (HSV), human immunodeficiency virus (HIV) and HTLV-1 to promote the formation of virological synapses (Vasiliver-Shamis et al., 2008; Nejmeddine and Bangham, 2010; Abaitua et al., 2013).

Among several viruses, HTLV-1 masters the art of remodeling the ECM, by forming a viral biofilm at the surface of infected cells (Pais-Correia et al., 2010). In this structure, numerous virions are embedded in the ECM of infected cells. Interestingly, spreading of virions in multiple collective entities have been described for many other viruses (Li et al., 2014; Sanjuán and Thoulouze, 2019), notably through transfer of previously accumulated virions in membrane invaginations or as aggregates, demonstrated for HIV. This collective spreading is hypothesized to provide a selective advantage by increasing virions stability, the number of virions delivered to target cells and thus global infectivity, and protection from immune response. However, virions accumulation in a viral biofilm has been clearly and convincingly demonstrated for HTLV-1 only (Li et al., 2014; Sanjuán and Thoulouze, 2019). Interestingly, the HTLV-1 biofilm appears to be very similar to bacterial biofilms (Thoulouze and Alcover, 2011), both consist in a microbial community embedded in an adhesive ECM. However, the similarities and differences between these bacterial and viral types of biofilms have been little discussed so far. In this review, we compare the molecular composition of bacterial and viral biofilms, and their pathophysiological impact on the host and on therapeutic strategies (summarized in Table 1). This comparative analysis of bacterial and viral biofilms highlights aspects of viral biofilms that are poorly understood, and shows how the understanding of bacterial biofilms may inspire future work on viral biofilms.

**Table 1:** Properties and functional roles of bacterial and viral infectious biofilms.

	Bacterial biofilms	Viral biofilms
Localization	Medical devices and host tissue	Host cell surface
Observation	*In vitro*, *in vivo*, *in situ*	*In vitro*
Molecular components	Exopolysaccharides, proteins and extracellular DNA	Exopolysaccharides, proteins
Process	Cyclic	Unknown
Functional roles	Chronic and recurrent infections Shield against antibacterial molecules Immune system escape Cancerogenesis Bacterial dispersion	Viral transmission, escape from plasmacytoid dendritic cells (pDCs) sensing

## Molecular Composition of Microbial Biofilms

Bacterial biofilms can be formed on abiotic and biotic surfaces. Indeed, a wide range of abiotic surfaces may be colonized by bacterial biofilms, such as implanted medical devices, surgery equipment, or river stones. Biotic surfaces may be host tissues, skin, or medical devices that have been rapidly coated by host ECM proteins such as fibrinogen or fibronectin in the blood plasma (Ratner, 2015; Khatoon et al., 2018). Surfaces have different chemical (charge and hydrophobicity) and mechanical properties (topography, roughness, and stiffness), which influence the speed of bacterial biofilm formation and strength of attachment. Typically, 5–35% of the biofilm volume is constituted by the microorganisms while the remaining volume is ECM. The ECM composition can vary greatly between biofilms, depending on the strains/species present, the shear forces experienced, the temperature and the availability of nutrients (Barbosa et al., 2015; Oliveira et al., 2015; Mizan et al., 2016). However, all bacterial biofilms share general attributes. They contain polymeric molecules constituting the ECM involved in intercellular adhesion. Matrix polymers include exopolysaccharides, proteins, teichoic acids, and molecules released by eukaryotic and prokaryotic dead cells, which are mainly negatively charged DNA, called eDNA after its release (López et al., 2010; Bjarnsholt et al., 2018).

Exopolysaccharides are the ECM components most frequently detected in biofilms. Most are long molecules, linear or branched, which can be homopolysaccharides or heteropolysaccharides consisting, for the latter, of a mixture of neutral and charged sugar residues. *Pseudomonas aeruginosa*, an important Gram-negative pathogen and a strong biofilm inducer, produces at least three distinct exopolysaccharides that contribute to biofilm development and architecture: Alginate, Pellicle (Pel), and Polysaccharide Synthesis Locus (Psl). Alginate is overproduced by mucoid strains isolated from lungs of cystic fibrosis patients, in which it is involved in the initiation of biofilm formation and in the mechanical stability of mature biofilms. Pel and Psl are involved in the establishment of biofilms in non-mucoid wild-type strains, which do not produce alginate. Pel is a glucose-rich polymer, essential for the formation of biofilms at air–liquid interfaces (called pellicles) and of biofilms that are attached to a surface, while Psl, a mannose-rich polymer, is involved in the adherence to abiotic and biotic surfaces and in the maintenance of biofilm architecture (Branda et al., 2005; Ghafoor et al., 2011). For the Gram-positive pathogen *Staphylococcus aureus*, polysaccharide intercellular adhesin (PIA), also known as poly-N-acetyl glucosamine, is a major exopolysaccharide found in biofilms. However, the presence of PIA is not universal and is for example, dispensable for the formation of staphylococcal biofilms (Mack et al., 1996; Brooks and Jefferson, 2014).

Unlike bacterial biofilm, the viral biofilm components that have been identified so far are not encoded by the virus itself nor synthesized by viral enzymes. Besides exploitation of existing cell pathways and functions for their own replication, viruses also alter physiological processes important for their dissemination. HTLV-1 can remodel several components of the eukaryotic ECM, enhancing their expression and modifying their spatial organization, thus leading to the formation of the viral biofilm (Thoulouze and Alcover, 2011). Extracellular components in which viruses are embedded in biofilm at the surface of the infected cells are made of complex polysaccharide assemblies, enriched in heparan sulfate, mannose and galactose exposing mainly the Thomsen-Friedenreich (TF) antigen, a desialylated Galβ1-3GalNAc glycosylated structure, and the tetrasaccharide sialyl-LewisX (sLex; Hiraiwa et al., 1997, 2003; Pais-Correia et al., 2010; Assil et al., 2019; Nakamura et al., 2019). Interestingly, Galectin-3 and Tetherin, have been found in viral biofilm and could be involved in the retaining molecules early steps (Pais-Correia et al., 2010; Nakamura et al., 2019). The viral biofilm is also composed of ECM components, like Agrin, HSPG, and Collagen IV (COL4A1 and COL4A2; Pais-Correia et al., 2010; Gross and Thoma-Kress, 2016; Millen et al., 2019). Moreover, Tarasevich et al. showed that the cell surface proteins CD4, CD150, CD70, CD80, and CD25 are also present in viral biofilm structures (Tarasevich et al., 2015). Although O-glycosylated surface receptors CD43 and CD45 do not seem to co-localize with viral particles, they were suggested to play an important role in the accumulation of viral biofilm-like structures on one pole of the HTLV-1-infected cell, facilitating contact with the target cell and efficient transmission of viral material (Mazurov et al., 2012).

Thus, polysaccharides and protein components are shared between bacterial and viral biofilm, while in contrast eDNA is exclusively found in bacterial biofilm. In addition, while direct interactions among bacteria have been documented in the bacterial biofilm, data on the interaction between viral particles in the viral biofilm are missing.

## Biofilm Formation and Pathogen Spreading

**Figure 1:**
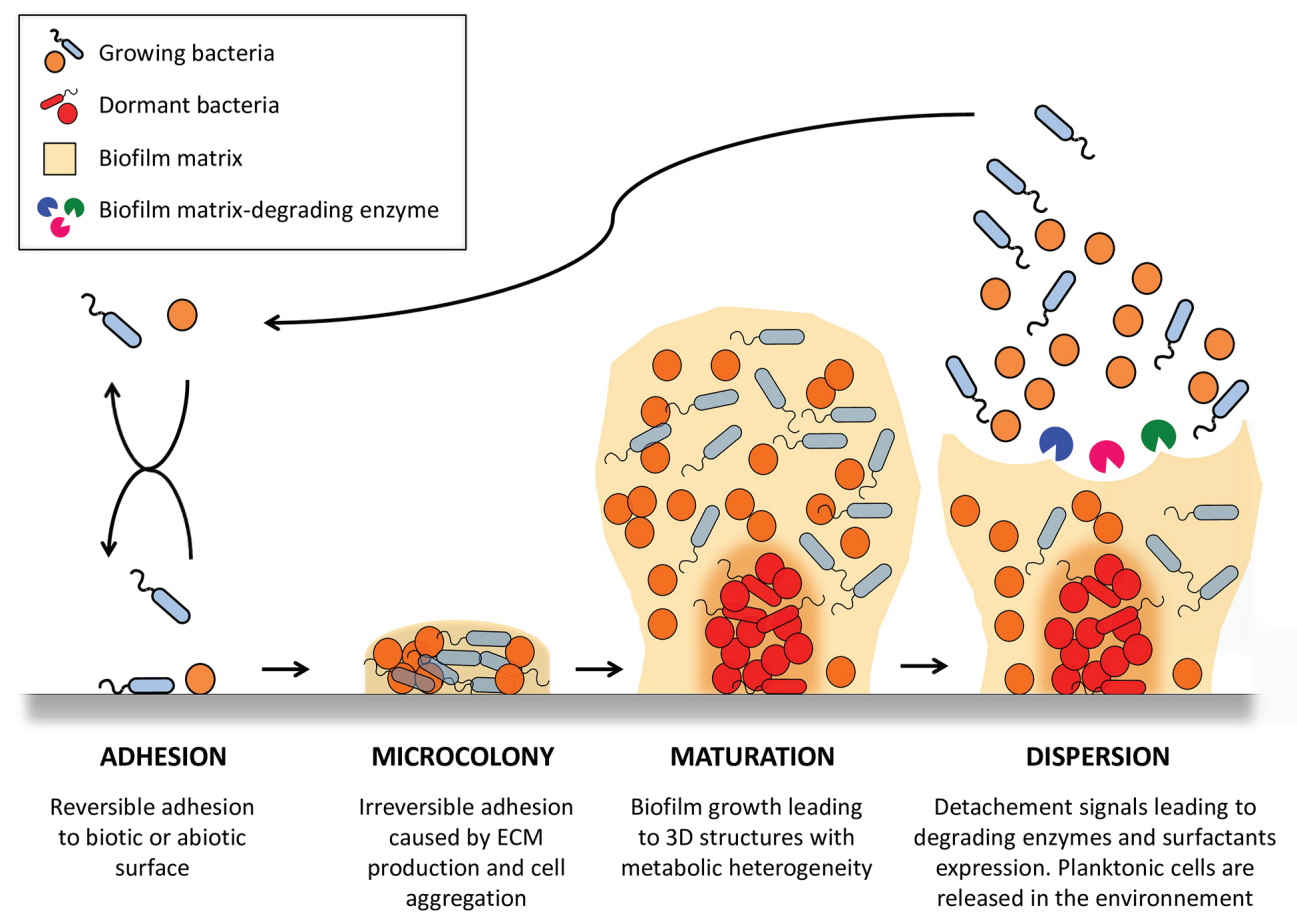
Stages of bacterial biofilm formation. One or more planktonic bacterial species adhere to an abiotic or biotic surface. Adhered bacteria grow as a multicellular community, forming microcolonies in which they proliferate and secrete matrix polymers. This microbial infrastructure results in the development of a 3D mature biofilm with heterogeneous physico-chemical conditions allowing the appearance of dormant state bacteria. Biofilms serve as bacterial reservoirs that are transmitted back to the environment to colonize new surfaces through biofilm detachment caused by intrinsic (biofilm matrix-degrading enzyme) and extrinsic (fluid shear) mechanisms. The figure was constructed using Servier Medical Art images.

Generally, bacterial biofilm formation is described as a cyclic process involving four main steps: adhesion, micro-colonies, maturation, and detachment (Figure 1). In the first step, bacteria have to adhere to a surface serving as a support (Armitano et al., 2013; Gagné-Thivierge et al., 2018). Mobile bacteria, such as *P. aeruginosa* and *Escherichia coli*, have a great advantage in this initial attachment, by utilizing flagella to overcome hydrodynamic and repulsive forces (O’Toole and Kolter, 1998; Wood et al., 2006; Liaqat et al., 2019). On abiotic surfaces, attachment depends on the surface properties of materials and bacteria, and involves hydrophobic interactions or Van der Waals forces (Renner and Weibel, 2011; Carniello et al., 2018). Wall components of several bacteria also serve as specific determinants for attachment. As examples, wall teichoic acids, lipoteichoic acids, and cell wall anchored proteins of staphylococci promote adhesion to abiotic surfaces (Vergara-Irigaray et al., 2008; Heilmann, 2011), while flagella, pili, or fimbria of *P. aeruginosa* play a role in surface attachment (Déziel et al., 2001; Vallet et al., 2001). When occurring on tissues or medical devices in the human body, adhesion is mainly governed by interaction of surface proteins from bacteria with human matrix proteins. In staphylococci, surface proteins belonging to the family of microbial surface components recognizing adhesive matrix molecules (MSCRAMMs) interact with fibronectin, fibrinogen, vitronectin, collagen, and other matrix molecules to allow adhesion of bacteria on host cell surfaces (Foster et al., 2014; Otto, 2018).

Once adhered to surfaces, bacteria form irreversible micro-colonies that are maintained through intercellular adhesion, leading to three-dimensional mature biofilm. This intercellular adhesion is allowed by the secretion of polymer molecules, which may be of carbohydrate, protein, or nucleic acid nature. In particular, the polyanionic charge of DNA is an important intercellular adhesion factor used to link molecules together. Of note, DNA release in biofilm ECM can be independent of bacterial lysis (Rajendran et al., 2015). Biofilm grows until it becomes a macroscopic “mushroom”-like structure. Such biofilms are not uniform and contain channels that are believed to be essential for providing nutrients to bacteria embedded deeper in biofilm layers. Indeed, bacteria are present in different physiological states (anaerobic, dormancy,…) depending on their location in the biofilm, and on the availability of substrates, oxygenation, pH, or exposure to metabolites present in the biofilm.

After biofilm maturation, isolated or aggregated bacteria can dissociate and end up in the bloodstream. This detachment stage allows colonization of other sites, leading to the appearance of “metastasis” of the infection. Bacterial release from the biofilm requires degradation of biofilm polymers by enzymes and surfactant molecules that reduce surface-bacterial interactions (Boles et al., 2005; Harmsen et al., 2010; Boles and Horswill, 2011; Kiedrowski et al., 2011; Otto, 2013). Several studies have demonstrated that this detachment process is subjected to quorum sensing, a regulatory mechanism that coordinates genes expression in the population (Boles and Horswill, 2008; Solano et al., 2014).

**Figure 2:**
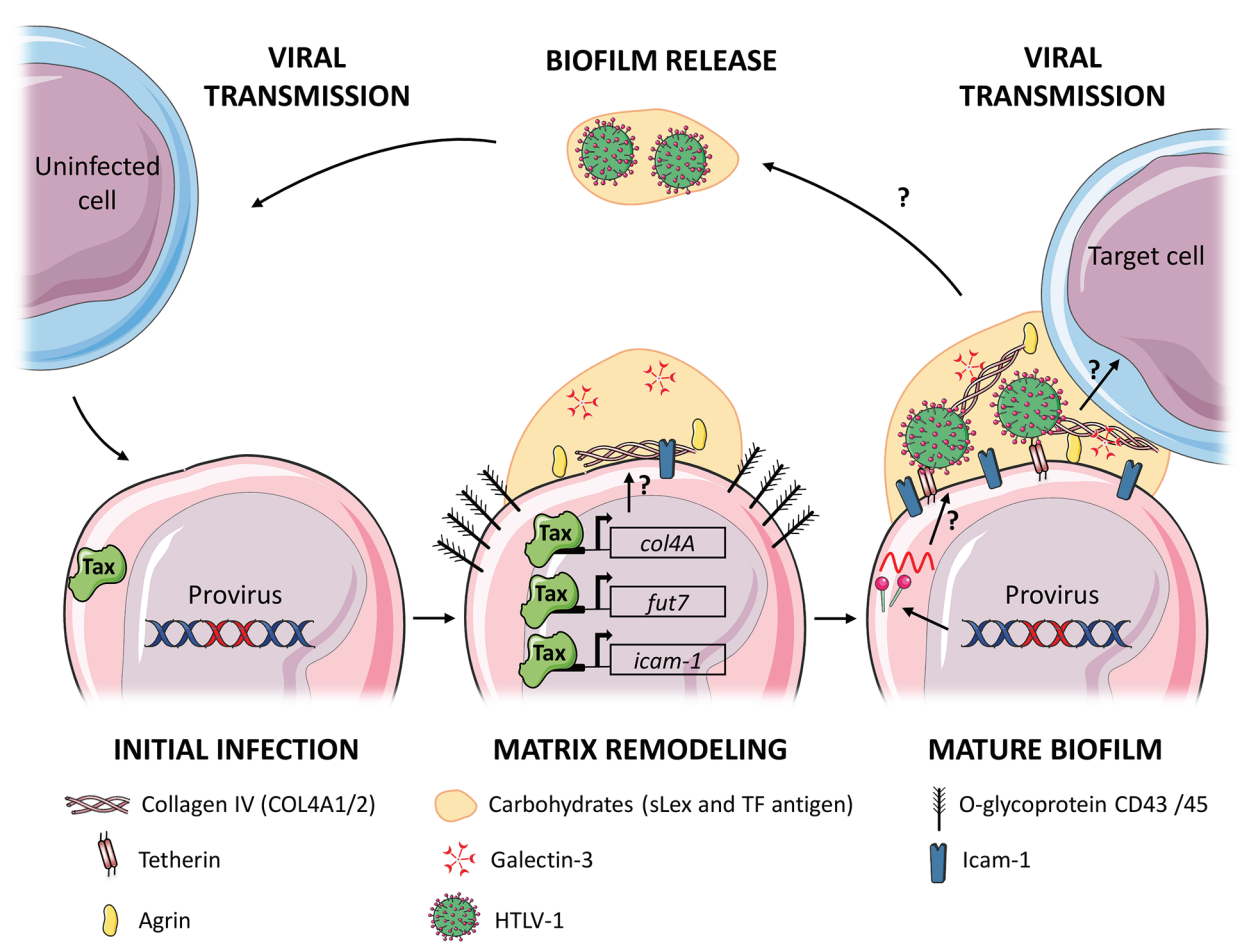
Proposed model for the stages of viral biofilm formation on human T-cell leukemia virus type 1 (HTLV-1) infected T cells. Following initial infection, viral expression (and in particular Tax expression) leads to remodeling of the extracellular matrix (ECM) components, such as carbohydrates (**sialyl Lewis X**), polymers components (Agrin and **Collagen IV**), linker proteins (Galectin-3 and Tetherin), and (Intercellular adhesion molecule-1, **ICAM-1**). Components whose expression is increased are underlined, and in bold when their expression is controlled by Tax. Production of viral particles embedded in the matrix forms the mature viral biofilm, which can be transmitted to a target cell by an unknown mechanism, or be released in the extracellular medium. The arrows with a question mark represent steps that remain to be determined. The figure was constructed using Servier Medical Art images.

Although the processes leading to the synthesis, stability, and regulation of bacterial biofilms have been widely studied, the kinetics and dynamics of ECM remodeling mechanisms leading to the formation of viral biofilm have not been investigated yet, but can be drawn from scarce literature data (Figure 2). Interestingly, the viral oncoprotein Tax induces the expression of genes involved in the synthesis of some HTLV-1 biofilm components, suggesting that HTLV-1 might directly drive the formation of this biofilm. It is for example, the case of Fucosyltransferase Fuc-T VII, which synthetizes sialyl Lewis X, but also of collagen IV (COL4A1, COL4A2; Hiraiwa et al., 1997, 2003; Pais-Correia et al., 2010) and of ICAM-1 (Hiraiwa et al., 2003; Fazio et al., 2019). Using transcriptome analysis, Millen et al. showed recently that *col4A1/2* genes are upregulated both in HTLV-1 infected cells and in Tax-induced cells, upon Tax-dependent transactivation of their promoter (Millen et al., 2019). Overexpressed COL4 proteins co-localized with the viral Gag protein in viral biofilm and were involved in viral transmission. In addition, Tax also induces expression of cell adhesion molecule-1 (CADM-1; Masuda et al., 2010), a protein involved in adhesion and cell infiltration, that could participate in the adhesiveness of the viral biofilm to target cells. Despite these studies, little is known about the role of Tax in formation of the viral biofilm. Thus, it would be interesting to study the formation of viral biofilm in HTLV-1 infected cells, in which viral expression is repressed but inducible such as MT-1 or ED-40515 cells (Mahgoub et al., 2018; Hong et al., 2020).

On the other hand, other components of the viral biofilm might not be induced directly by viral proteins, but rather indirectly, as a cellular response to infection. As an example, it is well established that Tetherin expression is induced in cells infected by various viruses (Evans et al., 2010), as a type I interferon (IFN-I)-induced innate immune response that restricts viral spread (Venkatesh and Bieniasz, 2013). Indeed, Tetherin expression dramatically inhibits the release of cell-free HTLV-1 particles by tethering them to the infected cell and to each other, without affecting viral protein expression (Jouvenet et al., 2009; Kuhl et al., 2011). Interestingly, while Tetherin restriction is counteracted by a variety of viral proteins (Evans et al., 2010), including Vpu from HIV-1 or Env from HIV-2 (Jolly et al., 2010), such a viral antagonism has not been described in the case of HTLV-1 infection, suggesting that Tetherin might not impact HTLV-1 transmission. Indeed, Tetherin knockdown has been shown to severely decrease HTLV-1 cell-free transmission but not cell-to-cell infection (Ilinskaya et al., 2013). Strikingly, cell-to-cell transmission analyzed in this study did not imply biofilm because viral transmission was measured using pseudovirus-expressing cells, thus devoid of HTLV-1 biofilm. Thus, the role of Tetherin in biofilm-based viral transmission still remains to be determined.

Knowledge is also missing on how viral biofilm is detached from the infected cell surface, and whether these released structures retain infectivity. However, this hypothesis is supported by the observation that removal of viral biofilm from the infected cells using heparin washes reduced HTLV-1 cell-to-cell transmission by 80% (Pais-Correia et al., 2010), and that purified HTLV-1 biofilm is the infectious entity responsible of viral transmission to target cells (Pais-Correia et al., 2010; Alais et al., 2015), probably more efficiently than transmission through the formation of a viral synapse (Igakura et al., 2003; Majorovits et al., 2008) or through cell-free viral particles, that are known to be poorly infectious *in vitro* and also rarely found *in vivo* (Li et al., 2008). Indeed, a ratio of around 10^6^ HTLV-1 virions is needed to infect one cell, while a ratio of 75 HIV virions is sufficient to infect one target cell (Derse et al., 2001). This lack of infectivity of cell-free virions has been linked to a reduced ability of Env protein to induce viral membrane fusion following isomerization of intersubunit disulfide-bound interactions observed in cell supernatant (Wallin et al., 2004; Li et al., 2008; Shinagawa et al., 2012). Thus, accumulation of virions inside the HTLV-1 biofilm might protect Env proteins from destabilization and could further maintain virus infectivity.

Alternatively, viral biofilm might favor cell-cell contacts through increased adhesiveness properties. This translates into close interactions among adhesion proteins (ICAM-1 and Lymphocyte function-associated antigen-1; LFA-1) and between viral and cellular proteins (Env and CD82/NRP-1). Those interactions were shown to trigger the polarization of the microtubule-organizing center (MTOC) in the infected cell, and as a result, the accumulation of viral proteins and viral RNA at the contact point, thus leading to the formation of a viral synapse and allowing viral particles’ budding at the synaptic cleft (Igakura et al., 2003; Majorovits et al., 2008). MTOC polarization in the infected cell upon cell-cell contact could also favor the delivery of extracellular vesicles (EV) to the target cell, a mechanism recently shown to increase the permissiveness of target cells (Pinto et al., 2019). In addition, polarization of the infected cells involves modification of actin polymerization, a process controlled by Tax through its induction of Gem, Fascin, and Collapsin response mediator protein-2 (CRMP-2; Masuda et al., 2010; Varrin-Doyer et al., 2012; Chevalier et al., 2014; Gross et al., 2016) and leading to increased cell-cell contacts and intercellular conduits (Chevalier et al., 2014; Gross et al., 2016). The latter are involved in the transfer of the p8 viral protein to target cells (Van Prooyen et al., 2010), which induces LFA-1 up-regulation. This in turn reinforces cell-cell adhesion through LFA-1 interaction with ICAM-1 (Van Prooyen et al., 2010; Malbec et al., 2011; Pinto et al., 2019).

It is unanimously accepted that Glucose Transporter 1 (GLUT-1), Neuropilin-1 (NRP-1), and Heparan Sulfate Proteoglycans (HSPG) are the three cell surface receptors involved in binding and entry of HTLV-1 cell-free virions in CD4+ T cells (Jones et al., 2011; Hoshino, 2012). Despite these advances, several questions remain unanswered, including their specificity and affinity for binding HTLV-1 present in biofilm and the precise role of each of these molecules during HTLV-1 entry in other types of cells. For example, HTLV-1 entry in dendritic cells (DC) seems to rely on endocytosis, as inhibition of micropinocytosis or clathrin-dependent endocytosis completely abolished HTLV-1 capture by immature and mature DC, as well as subsequent productive infection of immature DC (Rizkallah et al., 2017). Besides, the role of Glut-1 in HTLV-1 viral biofilm entry in plasmacytoid dendritic cell (pDC) was confirmed, but not that of NRP-1 (Assil et al., 2019). In addition, while the same study also confirmed the role of Glut-1 in productive infection of T-cells after contact with HTLV-1 viral biofilm, it failed to demonstrate the need of NRP-1, suggesting that the proposed interaction of HTLV-1 envelop with NRP-1 as prerequisite before interaction with Glut-1 (Lambert et al., 2009) might not be mandatory when virions are embedded in biofilm and not cell-free. However, whether viral biofilm is captured as a collective entity by the target cell, or whether individual virions are extracted from the biofilm before endocytosis is still unknown. Alternatively, one could hypothesize that because virions are associated with ECM in the viral biofilm, viral entry could be induced by interactions of ECM proteins such as integrins with their ligand, thus using regular routes of receptor recycling through endocytosis to enter in target cells. Then, interaction with Glut-1 could occur inside vesicles after the relief of ECM components. Indeed, this mode of entry is used by several bacterial species such as *S. aureus* and *Streptococcus pyogenes* (Rohde and Cleary, 2016; Josse et al., 2017), that expressed adhesions proteins able to bind fibronectin from ECM. Then, fibronectin interacts with the α_5_β_1_ integrin thus allowing the endocytosis of the complexed-bacteria in intracellular compartment.

Altogether, these results suggest that viral biofilm formation (i) might protect extracellular virions from Env protein instability, (ii) might increase cell-cell adhesiveness to favor cell-cell transmission, and (iii) might overcome the low infectivity of core particles, potentially through close interactions permitting transfer of viral proteins or cellular EV that might control target cell permissiveness.

## Microbial Biofilms and Their Role in Pathogenesis

### Biofilms in Immune Evasion Strategies

In an infectious context, immune response strategies are able to establish immediate and specific response due to effector mechanisms mediated by immune cells, recognition receptors, and several humoral factors such as complement components and antibodies. However, microbial biofilms can escape these host immune responses by shielding bacteria from immune effectors (Costerton et al., 1999; Roilides et al., 2015; Moser et al., 2017). Firstly, the bacterial biofilm provides a physical shield from antimicrobial peptides such as defensins, complement components, and opsonizing antibodies. Moreover, although leukocytes are able to effectively penetrate the biofilm, using the nutrient channels, they exhibit impaired phagocytosis and show a decreased ability to kill bacteria inside the biofilm (Leid et al., 2002; Scherr et al., 2015). Furthermore, biofilm polymorphonuclear neutrophils (PMN) clearance depends on the biofilm maturation state. Indeed mature biofilms are less sensitive to phagocytosis than “young” biofilms, which are more sensitive towards the attack by PMN (Günther et al., 2009). In addition, biofilm-embedded bacteria produce molecules that directly lyse immune cells and inhibit their function (Cheung et al., 2014; Da et al., 2017; Maali et al., 2018). For example, rhamnolipid, a quorum-sensing regulated virulence factor in *Pseudomonas* species, has been shown to lyse macrophages and neutrophils (Alhede et al., 2014). Interestingly, biofilm-derived products can actively suppress proinflammatory immune responses, as evidenced by the recruitment of myeloid-derived suppressor cells and macrophage polarization toward an anti-inflammatory state (Yamada and Kielian, 2019). Thereby, bacterial biofilms can subvert the host immune response by preventing immune detection, toxin production, impaired phagocytosis, and polarizing macrophages toward an anti-inflammatory state, which promotes biofilm persistence in an immune competent host.

Similar to bacterial biofilms, viral biofilms could also provide a physical barrier to prevent recognition by antibodies or immune cells. Assil et al. showed that the HTLV-1 biofilm regulates IFN-I production by pDCs (Assil et al., 2019). The presence of the TF antigen, a desialylated Galβ1-3GalNAc glycosylated structure at the surface of the infected cells, in which viruses are embedded in biofilm, reduces the IFN-I response of pDCs (Assil et al., 2019). Interestingly, the density of TF antigen at the surface of HTLV-1 infected cells positively regulates the ability of infected cells to transmit the virus and infect new target cells. Thus, TF antigen present at the surface of infected cells and in viral biofilm could both block innate immune responses while increasing viral dissemination, two processes that may facilitate the progression to HTLV-1-induced diseases. Alternatively, the presence of viral biofilm and specially highly glycosylated component of the ECM could physically protect virions from neutralizing antibodies although present at high levels (Hadlock et al., 1999) or could isolated virions from the plasma membrane and hence reducing direct binding of anti-HTLV-1 envelope antibodies to cell membrane and thus avoiding antibody-mediated cellular cytotoxicity (ADCC) or complement-mediated cytotoxicity.

### Escape From Therapeutics Strategies

Because bacterial biofilms constitute foci of microbial persistence, they play a major role in therapeutics failure. Indeed, they are a dreaded scourge in the field of antibiotic therapy (Stewart and William Costerton, 2001; Høiby et al., 2010; Sharma et al., 2019). Several studies have sought to compare the susceptibility of Gram-positive and Gram-negative planktonic and sessile bacteria to different antibiotics (Olson et al., 2002; Castaneda et al., 2016). The concentration required to kill sessile bacteria may be 1,000 times greater than that required to kill planktonic bacteria from the exact same strain. One of the first described mechanisms that account for this non-specific antibiotic tolerance is alteration of antibiotics diffusion within the biofilm. Indeed, the biofilm ECM acts as a physical barrier that can reduce penetration of antibiotics by congestion or by interaction with the electrical charges of the biofilm components (Stewart, 1996). Another mechanism of antibiotic tolerance within biofilms is related to the slower growth of bacteria deeply embedded in the biofilm. Indeed, the depth of the biofilm creates gradients of nutrients, oxygen, pH, and secondary metabolites that modify the bacterial environment. Thus, low amount of nutrients and oxygen are available when bacteria are far away from the biofilm surface. This, combined to high concentrations of metabolites, and more acidic pH, switches bacteria (than called persisters) into a dormant state, which renders them insensitive to antibiotics (Stewart and Franklin, 2008).

To overcome antibiotic resistance of bacterial biofilms, an alternative and very promising strategy is phagotherapy, which can be combined with antibiotic therapy (Sun et al., 2013; Zaidi et al., 2017; Misba and Khan, 2018; Moelling et al., 2018; Gordillo Altamirano and Barr, 2019). Being of small size, bacteriophages can circulate inside the biofilm using channels and reach bacteria laying deeper in the biofilm (Harper et al., 2014). Moreover, some phages produce enzymes that have the ability to deconstruct the ECM, thus increasing the accession of phages to bacteria for efficient infection (Chan and Abedon, 2015).

Compared to antibiotics treatment of bacterial infection, antiviral treatments have so far been poorly efficient for treating patients suffering from HTLV-1-induced diseases. Adult T-cell leukemia (ATL) patients are mainly treated with drugs that target infected cells regardless of their ability to express the virus (Miyazato et al., 2016), while HTLV-1-associated myelopathy/tropical spastic paraparesis (HAM/TSP) are treated with drugs reducing inflammation but not the proviral load (Futsch et al., 2017). Nevertheless, reducing the proviral load remains a promising strategy and screening of antivirals that target viral replication and viral dissemination are ongoing (Macchi et al., 2011; Pasquier et al., 2018; Barski et al., 2019).

The effect of antiviral treatment when viral transmission involves a viral biofilm is currently not known. However, it is clearly established that cell-to-cell spread authorized infection under adverse conditions such as antiretroviral therapy. Indeed, antiretroviral therapies did not impacted HIV infection when cell-to-cell transmission was used, while it was very efficient at reducing HIV infection using cell-free virions, no matter the classes of inhibitors used, including those targeting adsorption, entry, fusion, reverse transcription, and integration (Sigal et al., 2011; Agosto et al., 2015; Shimura et al., 2015). Although, the mode of HIV cell-to-cell transmission is thought to involved the formation of viral synapse (Pedro et al., 2019) one cannot exclude transfer of virus retained at the cell surface by Tetherin or T-cell immunoglobulin and mucin domain 1 (TIM-1; Perez-Caballero et al., 2009; Li et al., 2014; Roy et al., 2014), both expression are induced following HIV infection (Homann et al., 2011), in a process reminiscent of a viral biofilm, although the formal description of such HIV biofilm has to be demonstrated yet. Similar to bacterial biofilms, viral biofilms might play important roles in therapeutics failure. Therefore, development of therapeutic strategies that would specifically impede viral biofilm formation might optimize treatment efficiency in HTLV-1 carriers.

### Biofilm and Cancerogenesis

In addition to its role in the circumvention of the immune response and of therapeutic treatments, recent studies have highlighted the role of bacterial biofilms in cancerogenesis. To date, convincing evidence has linked the development of digestive tract cancers, and in particular colorectal cancer, with the presence of several key individual bacterial species, such as *Bacteroides fragilis*, *Streptococcus gallolyticus*, and *Helicobacter pylori* (Dejea and Sears, 2016; Raskov et al., 2018). It was suggested that formation of biofilm by these bacteria promotes an exacerbate inflammation combined to the secretion of toxic bacterial compounds. Moreover, alteration of the epithelial barrier function and modulation of the host metabolism might promote proliferation of epithelial cells (Johnson et al., 2015) and incidence of mutations, favoring cancerogenesis (De Weirdt and Van de Wiele, 2015; Li et al., 2017; Dejea et al., 2018). In the future, prevention and treatment of colorectal cancer patients might include microbiome and biofilm targeting.

HTLV-1 infection causes T-cell proliferation as a consequence of Tax and HBZ expression (Basbous et al., 2003; Higuchi and Fujii, 2009; Mesnard et al., 2015). ATL is characterized by pathologic amplification of transformed infected cells without the need of persistent viral production. One of the important clinical manifestations of ATL is the infiltration of leukemic cells from the blood into various organs, such as lymph nodes, spleen, lungs, and skin (Kaneko et al., 2015; Valencia and Moreno, 2017; Farès et al., 2018; Sharma et al., 2018). Strikingly, HTLV-1 viral production is not constant in ATL, but sporadic due to thin regulating mechanisms allowing evasion from host immune surveillance (Miyazato et al., 2016). Thus, one cannot directly infer the direct implication of the viral biofilm in leukemic cells infiltration, but the modification of ECM induced by HTLV-1 infection and the prerequisite for viral biofilm formation is certainly an important factor in leukemic cell infiltration. Accordingly, ECM modification is tightly linked to the migratory, polarization and infiltration capacity of cells, which are directly involved in the leukomogenesis of ATL (Kawaguchi et al., 2009; Nakachi et al., 2011; Futsch et al., 2018). Furthermore, the ability of cells to infiltrate tissues requires controlled cell migration, which is largely dependent on the regulatory protein Tax. As discussed above, Tax induces the overexpression of (i) CADM-1 and Gem, which are involved in the formation of lamellipods, (ii) Fascin in the formation of filopods, and (iii) CRMP-2 in cell polarization. HBZ is also involved through its ability to activate expression of the chemokine receptor CCR4 on T cells. Altogether, this increases migration and proliferation of infected cells, promoting the infiltration of leukemic cells into various organs and tissues (Giraudon et al., 1998; Masuda et al., 2010; Varrin-Doyer et al., 2012; Sugata et al., 2016). Enhancing tumor cells adhesiveness *via* ICAM-1/LFA-1 binding promotes interaction with endothelial cells, thus allowing their transendothelial migration (Mori et al., 2002). Once the endothelial barrier is crossed, tumor cells are confronted with the basal lamina and the interstitial tissue matrix (Poltavets et al., 2018). Many proteolytic enzymes degrade the components of the basal lamina and of the tissue ECM. Among these, matrix metalloproteinases (MMPs) can play an important role in tumor invasion and metastases (Bazarbachi et al., 2004; Gialeli et al., 2011). Interestingly, Tax induces overexpression of MMP-9 gelatinase B in cells infected with HTLV-1 (Mori et al., 2002), which could represent a key process involved in the invasiveness of ATL cells.

In addition to a decisive role in tissue infiltration of tumor cells, the ECM plays a key role in hampering the anti-tumor immune response, due to the isolation of malignant cells in the stroma, also called the tumor microenvironment (Lu et al., 2012; Manivannan et al., 2016). Extensively studied in epithelial cancers, the stroma surrounds the tumor cells clusters, and is composed of fibroblasts, cells of the vascular system, and effector immune cells with anti-tumor activity, in association with an ECM composed in particular of fibronectin and collagen fibers (Salmon et al., 2012). In these structures, the accumulation and mobility of infiltrating T and natural killer cells is very limited, which considerably reduces the specific antitumor response. The contribution of the tumor microenvironment in ATL development is not yet completely unraveled. However, using a transgenic and humanized mouse model, Vicario et al. showed that fibroblasts might enhance tumorigenesis of HTLV-1-infected and immortalized T cells, thus shedding light on the microenvironment contribution in ATL pathogenesis (Vicario et al., 2018). Although it is not denied that the direct or indirect ECM remodeling by viruses is involved in tissue infiltration and in the anti-tumor host’s immune response, the role of viral biofilm, which is constituted of ECM components involved in cancer pathogenesis, has never been investigated.

## Conclusion and Perspectives

HTLV-1 is almost exclusively transmitted during cell-cell contacts. The concentration of viruses at the viral synapse or in the viral biofilm allows the transfer of virions in a directional and extremely efficient way. This strategy limits the interactions of the virus with the environment, which could allow it to escape therapeutics strategies and immune responses. In comparison with bacterial biofilms, our understanding of the impact of the viral biofilm on the viral cycle is still very limited. In particular, whether the presence of virions in a biofilm modifies viral entry into the target cells is not completely understood. Is there viral diffusion inside the biofilm to reach the target plasma membrane and allow subsequent membrane fusion, or are viral particles and ECM endocytosed, then leading to fusion in endosomes? What are the molecular mechanisms that regulate these pathways? Moreover, is viral biofilm present on different HTLV-1 infected cell lines similar in terms of composition, structure, and topography, given that these infected cells have different infectivity and ability to escape to innate sensing (Alais et al., 2015; Assil et al., 2019)? Viral biofilm has only been reported in *in vitro* models using HTLV-1-infected cell lines (Pais-Correia et al., 2010; Nakamura et al., 2019). Further studies are needed to demonstrate the presence and the role of biofilm structures in *in vivo* models. One of the major difficulties is that the sites of viral transmission *in vivo* are not identified, probably because expression of HTLV-1 transcripts occurs in bursts or intermittently in infected cells, which considerably reduces the windows of observation. Furthermore, it is still very challenging to discriminate viral transmission using viral synapse or biofilm transfer given that these two mechanisms share several identical molecular players. Major questions also remain open about the role of viral biofilm during inter-individual viral transmission (Okochi and Sato, 1984; Kaplan et al., 1996; Hino, 2011) or its presence in biological fluids, such as maternal milk, blood, and saliva. Finally, analyzing whether other viruses transmitted by cell-cell contacts are also able to form a viral biofilm is of uttermost importance to build a generalized concept of viral biofilm.

## Author Contributions

All authors contributed to manuscript revisions, read and approved the submitted version. YM wrote the first draft of the manuscript.

### Conflict of Interest

The authors declare that the research was conducted in the absence of any commercial or financial relationships that could be construed as a potential conflict of interest.

## Abbreviations

ATL: Adult T-cell leukemia
CAM: Cell adhesion molecules
ECM: Extracellular matrix
eDNA: Extracellular DNA
HAM/TSP: HTLV-1 associated myelopathy/Tropical spastic paraparesis
HTLV-1: Human T-cell leukemia virus type 1
IFN-I: Type-I interferon
MMPs: Matrix metalloproteinases
MSCRAMMs: Microbial surface components recognizing adhesive matrix molecules
pDCs: Plasmacytoid dendritic cells
PIA: Polysaccharide intercellular adhesin
sLex: Tetrasaccharide sialyl-LewisX
TF: Thomsen–Friedenreich antigen.
